# Extensive Epidermoid Cyst and Breathing Difficulty

**DOI:** 10.1155/2015/826389

**Published:** 2015-06-09

**Authors:** Ciro Dantas Soares, Alberto Costa Gurgel, Francisco de Assis de Souza Júnior, Samila Neres de Oliveira, Maria Goretti Freire de Carvalho, Hanieri Gustavo Oliveira

**Affiliations:** ^1^Department of Oral Diagnosis, Piracicaba Dental School, Universidade Estadual de Campinas (UNICAMP), Avenida Limeira 901, P.O. Box 52, 13414903 Piracicaba, SP, Brazil; ^2^Department of Dentistry, Universidade Potiguar (UnP), Avenida Senador Salgado Filho 1610, 59056-000 Natal, RN, Brazil

## Abstract

Epidermoid cysts are common cystic lesions in the skin, ovaries, and testicles, but their occurrence in the oral cavity is uncommon. They consist of cysts delimited by a fibrous capsule without cutaneous annexes and are lined by stratified squamous epithelium. The differential diagnosis includes ranula, dermoid cysts, and lingual thyroid. Despite their benign presentation, these cysts can cause functional limitations, requiring special clinical attention for extensive lesions located in regions that preserve vital structures. This paper aims to report a case of epidermoid cyst in patient with swallowing and breathing difficulty, highlighting the clinical and surgical planning.

## 1. Introduction

Epidermoid cysts (epidermic cysts, EC) are common cystic lesions in skin, testicles, and ovaries derivates of ectoderm-lined inclusion. They comprise less than 0.01% of all oral cavity cysts and their occurrence in the floor of the mouth with respiratory complications has not been reported [1, 2].

Histologically, EC are bounded by fibrous capsule and are composed of an epithelium which is flattened and contains a granular layer of keratohyalin granules. Absence of hair follicles, sebaceous glands, and apocrine sweat glands (skin appendages) in capsule of these cysts helps differentiate between dermoid cysts [3]. The dermoid and epidermoid cysts are indistinguishable in the clinical and radiographic exams and require microscopic analysis for differentiation [4].

Etiology of EC remains unknown and the most accepted theory is the reactivation of the remaining ectoderm trapped in the 1st and 2nd pharyngeal arches. However, accidental or traumatic inclusion of epithelial tissue in deep structures of the dermis or submucosa may be associated with pathogenesis of epidermoid cysts [5, 6].

The epithelial cells malignant transformation of these cysts has been reported but is rare [7, 8]. Extensive lesions located at regions that preserve vital structures may cause functional limitations, requiring special clinical attention. Early diagnosis of epidermoid cysts permits good functional and aesthetic results. The need for interaction in a multidisciplinary team must be assessed [9, 10].

This paper aims to report a case of an extensive epidermoid cyst on buccal floor, with emphasis on the importance of image diagnosis (Cone-Beam Computed Tomography) for treatment planning.

## 2. Case Report

**Figure 1:**
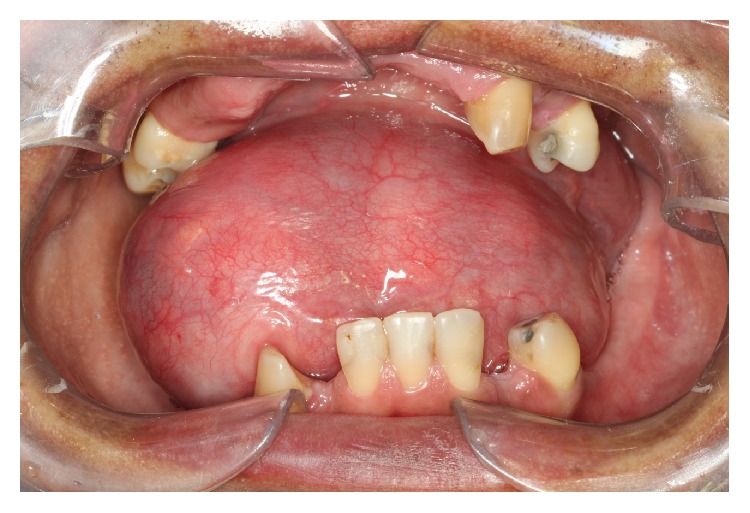
Clinical aspect of mass in floor of the mouth, asymptomatic. The patient related difficulty breathing and swallowing.

A 45-year-old male patient presents with extensive mass in the buccal floor, with limitation in mouth opening and speech associated with dysphagia and dyspnea. The period of evolution of lesion was unknown. The clinical examination revealed an expansive mass, asymptomatic, exophytic, and no history of associated trauma, and fluctuated upon palpation (Figure 1). The lesion surface had normal-appearing overlying mucosa. The clinical diagnosis was ranula, dermoid cyst, or epidermoid cyst.

As patient reported swallowing and breathing difficulty, additional hematological examinations were performed, which showed normal range values. For surgery planning purposes and for observed relationship with soft tissues and other anatomical structures, a CT scan was performed showing the dimensions of the lesion, as well as confirming the hypothetical diagnosis of the internal liquid contents. Aspiration puncture demonstrated content material friable and white. Surgical planning included complete lesion excision. After this the specimens were removed and were sent for anatomical pathologic evaluation.

The ovoid cystic mass was macroscopically observed to be opened and without any content. It was measured to be 5.0 × 3.0 × 0.2 cm and it had brown pigmentation with a few whitish areas. Microscopic examination revealed a cystic cavity with a capsule composed of dense fibrous connective tissue, lined by stratified squamous epithelium resembling epidermis (Figure 4(a)). There were no skin appendages in the capsule. The lesion contents were represented by concentric blades of orthokeratin. A breach on the cyst wall with chronic granulomatous inflammation and multinucleated giant cells was also observed (Figure 4(b)), including keratin, being the final diagnosis of a ruptured epidermoid cyst, with granuloma to the foreign body (keratin).

## 3. Discussion

The epidermoid cysts (EC) have uncertain etiology and may be formed from reactivation of epithelial remnants entrapped during midline closure of the bilateral first and second branchial arches. Another probable cause is accidental introduction of epithelium in the subcutaneous tissues or in the submucosa after extraction of a third molar, for example, [6, 11, 12].

They affect mainly male patients and are uncommon during childhood or puberty. Their occurrence in the oral cavity is rare and represents less than 0.1% of all oral cysts [11, 13]. Despite the benign course of EC, these lesions may reach masses of large volume due to production of keratin within the cyst, as an attempt to balance the osmotic pressure, and were related to its malignant transformation [7, 8].

EC have different diagnosis such as infectious lesions of the salivary glands, ranula, dermoid cyst, lipoma, lingual thyroid, and thyroglossal duct cyst [3, 4, 12, 14–16]. In the present case the hypothesis of diagnosis was ranula, dermoid cyst, and EC (Figure 1). The patient reported swallowing and breathing difficulty, probably occasioned for posterior expansion of the lesion in submandibular space.

Computed tomography (CT) is a reliable assessment of lesion extension to deeper structures when diagnosing or evaluating the submandibular space. CT permits visualization of the differences in densities of hard and soft tissues thus optimizing the diagnosis and it guides the surgeon for a more efficient treatment plan and enables visualizing the relationship of the lesion with muscles, salivary glands, and other tissues [2, 17, 18]. The submandibular space contains submandibular salivary gland; and the lingual nerve, artery, and vein and these structures are necessary for the physiology of oral cavity.

**Figure 2:**
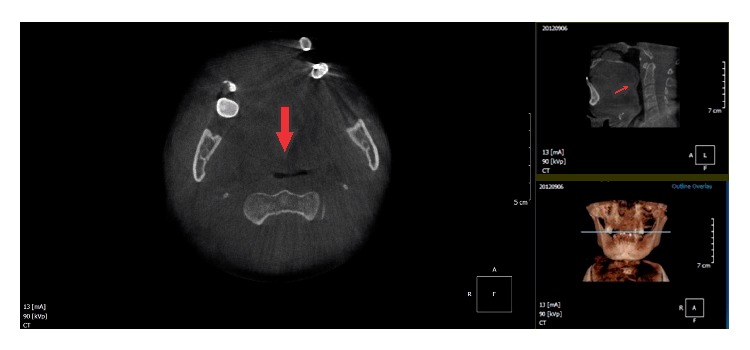
Cone-Beam Computed Tomography. Axial section showing extensive lesion on the buccal floor (in the sublingual space), demonstrating hypodense areas and almost airway obstruction (red arrows).

In this case for safe surgical approach,was observed the depth of the lesion in the submandibular space. Also it became evident that the lumen of the lesion is of low density, assisting in the appropriate development of a clinical diagnostic. Expansion of the lesion was confirmed with a reduction of nasopharynx space, observed in CT images (Figure 2).

**Figure 3:**
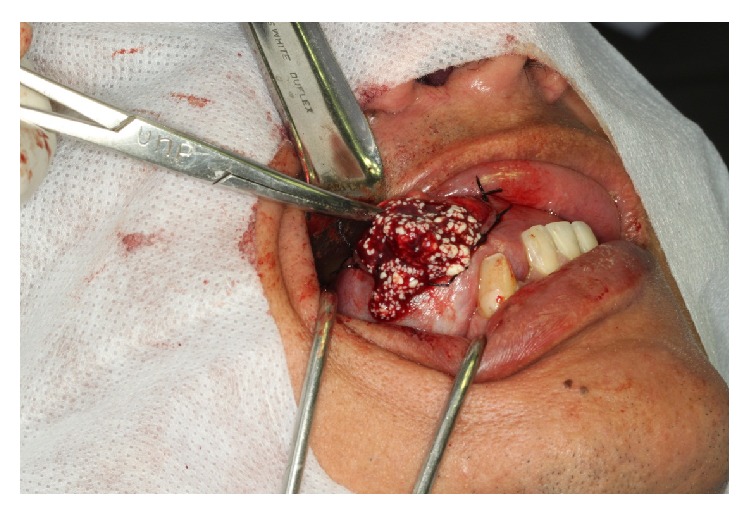
Aspect of contents of lesion during surgical excision: material white, friable, and compatible with orthokeratin.

The imaging examinations also allow selecting the surgical approach: intra- or extraoral. In our case we decided to use the intraoral approach, despite a large dimension cyst, which presented superficial involvement. During surgical procedures the contents of the cysts revealed friable and white material compatible with keratin (Figure 3).

**Figure 4:**
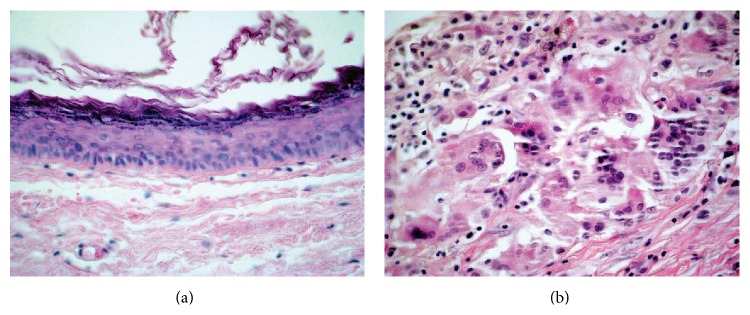
Microscopic findings: (a) the lining of the cyst is composed of an epithelium which is flattened and contains a granular layer of keratohyalin granules, Haematoxylin and Eosin, 100x, and (b) foci of rupture and keratin exposed to the adjacent capsule and reaction composed of macrophages and foreign body giant cells (for keratin exposed), Haematoxylin and Eosin, 400x.

The dermoid cyst can occur in the skin, ovary, testicles, and other regions of the body always related to the midline, because it matches teratomatous injuries. In the capsule, microscopic examination showed the presence of skin appendages in the cyst wall, in addition to the stratified squamous lining. The gonadal lesions often exhibit other mature tissues such as cartilage, bone, fatty tissue, and nerve tissue, as well as skin structure [19]. According to the microscopic findings, the present case is an epidermoid cyst with foci of rupture and keratin exposed to the adjacent capsule with foreign body (keratin) and reaction composed of macrophages and foreign body giant cells (Figure 4).

A total lesion excision was the recommended treatment, and the recurrence is unusual. The risk of malignancy of these cysts is rare, but there are reports on dental literature. The etiology and pathogenesis of the EC require more conclusive studies [20].

As mentioned, epidermoid cysts are benign lesions; however, they may have large dimensions and cause physiological complications including swallowing and breathing difficulty. The computed tomography is a method efficient for assessing the relationship with the adjacent anatomical structures and planning of surgical approach.
